# miR-328-5p Induces Human Intervertebral Disc Degeneration by Targeting WWP2

**DOI:** 10.1155/2022/3511967

**Published:** 2022-09-29

**Authors:** Jing Yan, Lun-Gang Wu, Ming Zhang, Tao Fang, Wei Pan, Jia-Li Zhao, Quan Zhou

**Affiliations:** ^1^Department of Orthopaedics, The Affiliated Huai'an Hospital of Xuzhou Medical University and The Second People's Hospital of Huai'an, Huai'an, Jiangsu 223002, China; ^2^Department of Orthopedics, Zhongda Hospital, School of Medicine, Southeast University, Nanjing, Jiangsu 210009, China; ^3^Department of Orthopedics, The First People's Hospital of Changshu, Jiangsu 210009, China

## Abstract

Intervertebral disc degeneration (IDD) development is regulated by miRNA, including inflammatory reactions, cell apoptosis, and degradation of extracellular matrix. Nucleus pulposus cells apoptosis has a absolute influence in the development of IDD. This experiment explores the mechanism of miR-328-5p regulating IDD. Through the analysis of miRNA and mRNA microarray database, we screened the target genes miR-328-5p and WWP2. We verified the expression of miR-328-5p, WWP2, and related apoptotic genes in normal and degenerative nucleus pulposus tissues by qRT-PCR. The expressions of WWP2, Bcl-2, and Bax were detected by qRT-PCR and western blot after transfection to nucleus pulposus cell. The nucleus pulposus cell proliferation and apoptosis after transfection were confirmed by CCK8 and flow cytometry. Luciferase reporter assay and bioinformatics analyzed the targeting relationship between miR-328-5p and WWP2. Firstly, the qRT-PCR experiments confirmed the significant increase of miR-328-5p expression, while significant reduction of WWP2 in a degenerative tissues compared to the normal tissues. Surprisingly, miR-328-5p expression was positively, while that of WWP2 negatively correlated with the degeneration grade of IDD. And we also identified the high expression of Bax and Caspase3, while low expression of Bcl-2 in a degenerative tissues. After miR-328-5p mimic transfected into nucleus pulposus cell, qRT-PCR and western blot confirmed that WWP2 and Bcl-2 expressions were downregulated, while Bax and Caspase3 expressions were upregulated, and the same results were obtained by knocking down WWP2. CCK8 and flow cytometry confirmed that miR-328-5p inhibited the proliferation and induced apoptosis of nucleus pulposus cells. WWP2 is a target gene of miR-328-5p by bioinformatics and luciferase reporter assay. In summary, miR-328-5p targets WWP2 to regulate nucleus pulposus cells apoptosis and then participates in the development of IDD. Furthermore, this study may provide new references and ideas for IDD treatment.

## 1. Introduction

With the increasing incidence of low back pain (LBP), it has become the most important trigger to disability worldwide, which has brought a tremendous economic pressure [1–3]. The cause of LBP is very complex. And some known factors affect the advancement of LBP include genes, age, and lousy living habits (such as occupation, smoking, trauma, and mechanical loading) [4, 5]. Furthermore, it is believed that the main cause of LBP is IDD [6–8]. As a bridge between adjacent vertebral bodies, intervertebral disc includes the nucleus pulposus (NP), annulus fibrosus, and the cartilaginous endplate [9]. The most important pathological feature of IDD is the apoptosis of nucleus pulposus cells [10–13]. The apoptosis of nucleus pulposus cells triggers the progress of IDD [14–16], and this affects clearly the disc structure balance. Some studies suggested that abnormal apoptosis is associated with degenerative diseases such as osteoarthritis, IDD, and cancer [17–19].

However, the current treatment for IDD is limited to symptomatic intervention and cannot completely improve the prognosis of the disease [20]. Many studies confirmed that some regulatory genes have an essential impact in the incidence and development of IDD, such as microRNA (miRNA). The miRNA are single-stranded noncoding small RNA with 18 to 24 bp nucleotide sequences, which participates in regulating the cell proliferation and apoptosis [21–25]. And miRNA can negatively regulate the posttranscriptional gene expression in different species by either inhibiting mRNA translation or promoting mRNA degradation [25, 26]. Previous studies have also found that miRNA affects the progress of IDD by facilitating inflammatory response, cell apoptosis, and degradation of extracellular matrix [27]. Furthermore, miRNA has contributed to cardiovascular disease, cancer, leukemia, and skeletal muscle diseases [28]. Therefore, further research on the mechanism of miRNA regulation of IDD may lead to new therapeutic directions.

Some studies have found miR-328-5p is atypically expressed in lung cancer, breast cancer, and other tumors. In addition, the researcher indicated miR-328-5p was related with the proliferation and apoptosis of cancer cells [29–32]. Furthermore, studies have shown dissimilarity expression of WWP2 in various diseases such as oral cancer, endometrial cancer, ovarian cancer, glioma, and lung cancer by regulating cell apoptosis [33–38]. Both miR-328-5p and its target gene WWP2 can affect cell proliferation and apoptosis, and the mechanism of miR-328-5p mediating WWP2 regulating IDD has not been reported. This series of studies aims to research the mechanism of the above regulatory pathways and whether they are involved in the formation of IDD by inducing nucleus pulposus cell apoptosis.

## 2. Materials and Methods

### 2.1. Clinical Sample Collection

We obtained 20 human NP samples via surgical discectomy. Surgical indications: (1) failure of conservative treatment and (2) progressive neurological deficits. Patients were excluded with isthmus or degenerative spondylolisthesis, ankylosing spondylitis, and diffuse idiopathic hyperostosis. According to T2-weighted midsagittal pfirrmann disc degeneration grading criteria [39].Grade I-II are normal intervertebral discs, and grade III-V are degenerative intervertebral discs.  Table 1 presents the clinical features of patient.

### 2.2. Isolation and Culture of NP Cells

The NP tissue was separated from the intervertebral disc, made into 2-3 mm^3^ sections under the microscope in aseptic conditions, and washed three times with PBS. Then prepared tissues were digested with 0.25 mg/mL type II collagenase (Invitrogen; Thermo Fisher Scientific) at 37°C for 12 h in Dulbecco's Modified Eagle Medium (GIBCO, NY, USA). Then NP cells isolated from NP tissues by trypsin were placed in DMEM/F12 incubated at 37°C with 5% CO_2_. The second generation of cells was chosen for the following experiment.

### 2.3. Transfection of NP Cells

The miR-328-5p mimic/inhibitor, mimi/inhibitor control, WWP2 siRNA, and siRNA control were derived from Sangon (Shanghai, China). In our study, miR-328-5p mimic/inhibitor promote/inhibit the expression of miR-328-5p, and mimic/inhibitor control were used as control groups, respectively. WWP2 siRNA inhibits WWP2 expression in the NP cells. According to the reagent instructions, RNA transmate was used to transfect the NP cells (Sangon, Shanghai, China). The cells were collected 48 hours later for subsequent experiments.

### 2.4. qRT-PCR

General RNA was obtained from NP tissue or cultured NP cells using TRIZON (TianGen, Beijing, China) according to reagent instructions. We quantified miRNA and mRNA expression levels in NP tissues or cells by LightCycler 480 II (Roche Diagnostics, Indianapolis, USA). The PCR reaction system of 20 ul contained 10 ul Universal SYBR Green Fast qPCR Mix, 2 ul cDNA, 0.8ul primers, and 7.2 ul nuclease-free water. The reaction conditions: 95°C for 15 min, 40 cycles for 10 s at 95°C, and 60°C for 20s. The experiment was performed three times. Experiment is based on U6 and *β*-actin [40]. Gene expression was measured using the 2^−△△Ct^. Primers are as follows: miR-328-5p forward: AACGAGACGACGACAGAC, reverse: GGGGGGGCAGGAGGGGCTCAGGG; WWP2 forward: GAGATGGACAACGAGAAG, reverse: CTCCTCAATGGCATACAG; Bcl-2 forward: CAGCTGCACCTGACGCCCTT, reverse: GCCTCCGTTATCCTGGATCC; Bax forward: CCCGAGAGGTCTTTTTCCGAG, reverse: CCAGCCCATGATGGTTCTGAT; Caspase3 forward: ATGGTTTGAGCCTGAGCAGA, reverse: GGCAGCATCATCCACACATAC; U6 forward: CTCGCTTCGGCAGCACA, reverse: AACGCTTCACGAATTTGCGT; *β*-actin forward: AGGGGCCGGACTCGTCATACT, reverse: GGCGGCACCACCATGTACCCT.

### 2.5. Western Blot

The protein was obtained with RIPA and BCA (Beyotime, Shanghai, China) measures protein concentration. The NC membrane was blocked with 5% skimmed milk at room temperature for 2 h, then washed three times with TBST and added overnight to primary antibody at 4°C. The primary antibodies: anti-WWP2 antibody (Proteintech, Wuhan, China), anti-Bax antibody (Cell Signaling, Danvers, MA, USA), anti-Bcl-2 antibody (Cell Signaling, MA, USA), anti-Caspase3 antibody (Cell Signaling, MA, USA), and anti-beta-actin antibody (Proteintech, Wuhan, China). After primary antibody incubation, the NC film was washed three times with TBST and addted to goat anti-rabbit or mouse antibody (Vicmed, Xuzhou, China) for 2 h at room temperature in the dark. Beta actin was selected as the internal control [40].

### 2.6. CCK8

The cultured NP cells were transferred on 96-well plates by 2 × 10^4^ cells/well, and added with miR-328-5p mimic/inhibitor, and miR-control. Subsequent to the incubation, the cell culture medium was changed at 0 h, 12 h, 24 h, and 36 h of each well. 10 ul CCK8 reagent and 90 ul DMEM were added into every well and incubated for another 2 h at 37°C by the CCK8 kit (Vicmed, Xuzhou, China). The optical density was measured at 450 nm, and the experiment was repeated three times for each group.

### 2.7. Flow Cytometry

The detection of NP apoptosis was carried out by the flow cytometry instructions (KeyGEN, Nanjing, China). First, the transfected NP cells were separated in 0.25% trypsin (without EDTA) (Vicmed, Xuzhou, China). After rinsing the cells twice with PBS, 1 × 10^5^ cells were collected by centrifugation for 5 min at 2000 rpm. It is essential to rinse off as much residual trypsin digestive fluid as possible. After suspension of 500 ul binding buffer, each centrifugation tube was added 5 ul Annexinv-APC and 5 ul PI. The samples were thoroughly mixed, and the reaction was carried out at room temperature in the dark for 5-15 min. Finally, flow cytometry was used for observation and detection. Three experiments are required.

### 2.8. Luciferase Reporter Assay

In order to construct wild or mutant-type expression vectors, the WWP2 3′-UTR binding to miR-328-5p was inserted into the GV272 vector. Then, NP cells were added with wild type (Wt) or mutant type (Mut) WWP2 3′-UTR reporter plasmid and miR-328-5p mimic. Luciferase enzyme activity was detected according to Promega (Madison, WI, USA) reagent instructions after transfection 48 h. The luciferase enzyme activity was normalized to renilla luciferase activity. And western blot was used to detect WWP2 protein expression. The experiment was performed three times.

### 2.9. Statistical Analysis

Statistical was analyzed by the SPSS 26 (SPSS, Chicago, USA). GraphPad Prism 8.4 (GraphPad Software, CA, USA) was used for graphical representation. Mean ± SD was used to analyze the experimental data. *t*-test or one-way ANOVA was used for inter-group data analysis. Pearson's test was used for correlation analysis. *P* < 0.05 indicates statistical difference.

### 2.10. Ethics Statement

The Ethics Committee of Huai'an Affiliated Hospital of Xuzhou Medical University approved this study. Human NP tissue samples were obtained from patients who underwent surgery in Huai'an Affiliated Hospital of Xuzhou Medical University from September 2020 to April 2021. Meanwhile, the patients' written consent was informed, and the tissue samples were obtained during the operation.

## 3. Results

### 3.1. Assessment of Differentially Expressed miRNAs

**Figure 1:**
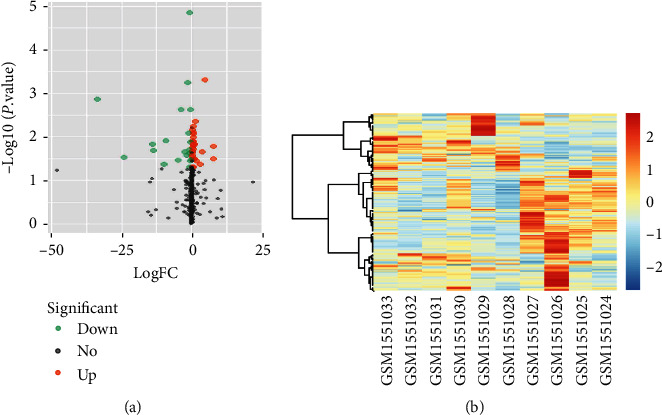
The screening of miRNAs expressions in normal and degenerative nucleus pulposus tissues. (a) a volcano plot demonstrates the upregulation and downregulation of different miRNAs expression by |logFC| > 1, |P.V value| < 0.05 as selection criteria. (b) heatmap for hierarchical clustering of selected miRNA expression in the tissue sample.

A volcanic map of the dataset (GSE63492) showed that some miRNAs are differentially expressed between normal and degenerated nucleus pulposus tissues among the 31 miRNAs, hsa-miR-328-5p, and hsa-miR-183-3p expression were upregulated. And 21 miRNAs expression, including miR-486-5p, hsa-miR-486-5p were downregulated (Figure 1(a)). Heatmap figure shows that individual miRNA expressions differed significantly (Figure 1(b)).

### 3.2. Assessment of Differentially Expressed mRNAs

**Figure 2:**
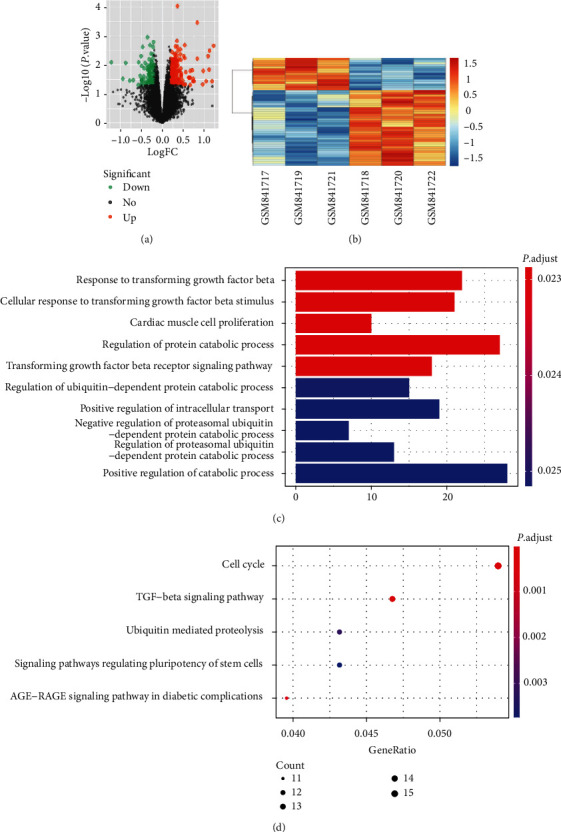
Assessment of different mRNAs expressions in normal and degenerative nucleus pulposus tissues. (a) a volcano plot of mRNAs expression by |*l*ogFC| > 1, |P.V value| < 0.05 as selection criteria. (b) heatmap for hierarchical clustering of selected mRNA expression. (c and d) GO, and KEGG functional annotations were performed on the differentially screened mRNAs, respectively. The bar chart represented GO enrichment analysis, while the bubble chart represented KEGG enrichment analysis.

The volcano map analysis database (GSE34095) obtained a pairwise comparison of mRNA expression between normal and degenerative nucleus pulpous tissues. The experiment identified 348 differentially expressed upregulated genes such as TGFBI and PDGFC, while 260 downregulated genes such as WWP2 and MPST (Figure 2(a)). Further, the stratified clustering analysis of intervertebral disc dataset using heatmap revealed differences in the expression of some genes (Figure 2(b)). GO including molecular function, cellular component, and biological process, and KEGG enrichment analysis was performed for differentially expressed mRNA using R language (Figures 2(c) and 2(d)).

### 3.3. Interactions between miRNA and mRNA

**Figure 3:**
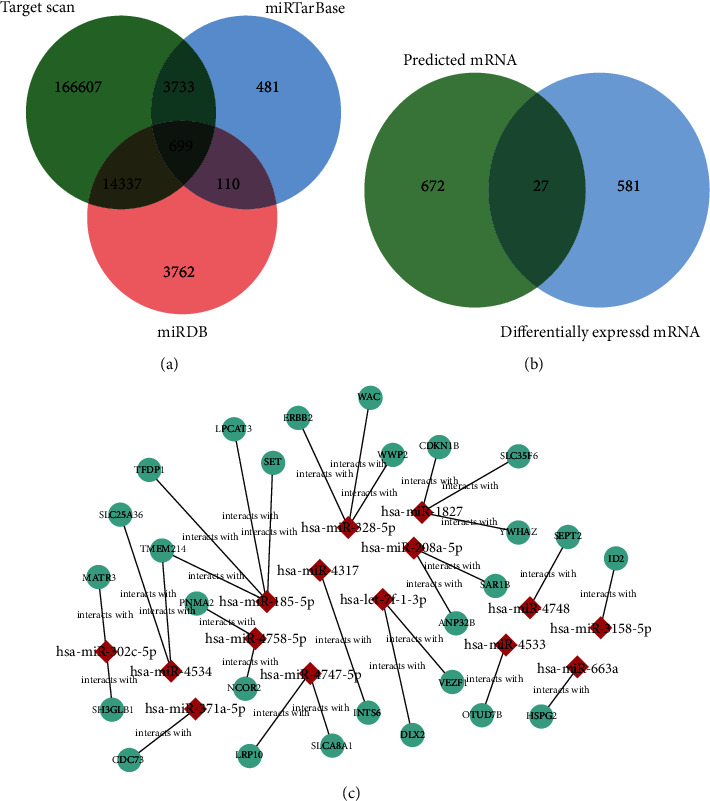
Interaction between differential miRNAs and their downstream target gene mRNAs: (a) the intersection of three datasets, (b) 27 different mRNAs, and (c) cytoscape analyzed the network diagram.

In order to analyse the relationship between mRNAs and miRNA, we go through miRTarBase (https://www.mirbase.org), TargetScan (http://www.targetscan.org/), and miRDB (http://mirdb.org/index.html) database to predict the miRNA target gene. The intersection of threedatasets was obtained by the Venn diagram representing 35 miRNAs and their downstream 699 target gene mRNAs (Figure 3(a)). And 27 different mRNAs containing WWP2 were obtained by predicting intersection mRNAs via Venn diagram (Figure 3(b)). Furthermore, cytoscape analyzed the network diagram of 27 differential mRNAs and their upstream miRNAs (Figure 3(c)).

### 3.4. Correlation and Modulation of miR-328-5p and WWP2 Expression

**Figure 4:**
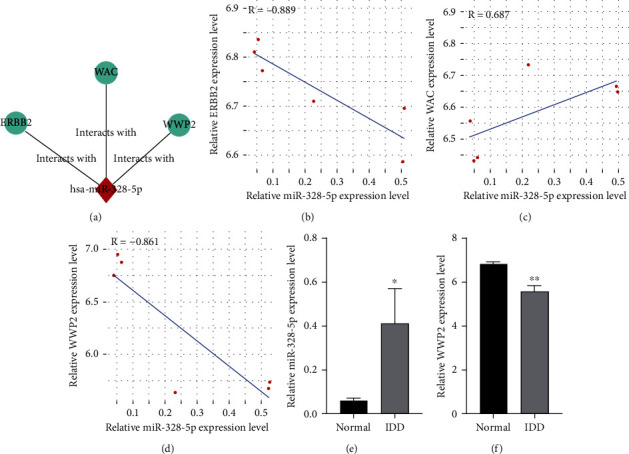
Correlation and differentially expression of miR-328-5p and WWP2. (a) the network diagram between miR-328-5p and its downstream genes. (b, c, and d) the correlation between miR-328-5p and its target genes. (e and f) significant differences in miR-328-5p and WWP2 expression.^∗^*P* < 0.05, ^∗∗^*P* < 0.01.

Cytoscape was performed to visualize the network diagram between miR-328-5p and its downstream genes (Figure 4(a)). Further, we confirmed the correlation between the miR-328-5p and its target genes by gene chip data and analyzed it using Pearson correlation analysis (Figures 4(b), 4(c), and 4(d). The microarray data expression of miR-328-5p and WWP2 genes were statistically significant (Figures 4(e) and 4(f)). The most important pathological feature of IDD is the apoptosis of nucleus pulposus cells [10–13]. Bioinformatics analysis shows that WWP2 is a downstream target gene of miR-328-5p. The conditions of target genes we selected were as follows: high connectivity of network diagram, negative correlation between miRNA and its downstream target mRNA, regulate cell apoptosis, and at the same time, relevant literature was reviewed to understand the function of miRNA and mRNA, so we selected miR-328-5p-WWP2 pathway as our research object. We hypothesize that miR-328-5p regulates IDD development by mediating WWP2.

### 3.5. Differentially Expressed of Relevant miRNA and mRNA in Degenerative and Normal Nucleus Pulposus Tissues

**Figure 5:**
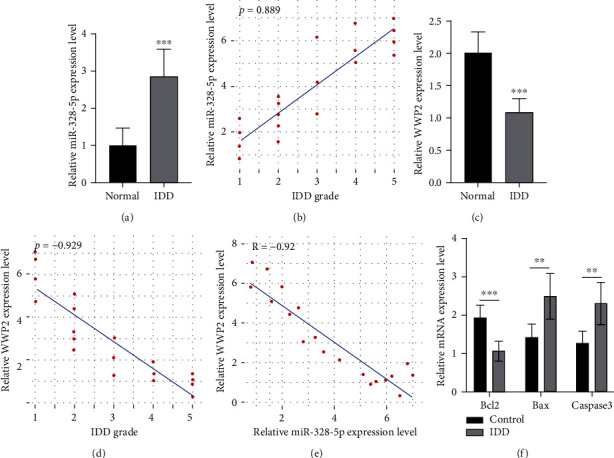
Differential expression of relevant miRNA and mRNA. (a) miR-328-5p expression difference. (b) Correlation between miR-328-5p gene expression with IDD grade. (c) WWP2 gene expression difference. (d) Correlation between WWP2 gene expression with IDD grade. (e) Correlation between miR-328-5p and WWP2. (f) Bcl-2, Bax and Caspase3 gene expression. ∗∗*P* < 0.01, ∗∗∗*P* < 0.001, degenerative VS normal nucleus pulposus tissues, *n* = 10.

Experiment confirmed that miR-328-5p gene expression was upregulated (*P* < 0.01, Figure 5(a)). Spearman's correlation found that miR-328-5p expression was positively correlated with IDD grade (*P* = 0.889, *P* < 0.001, Figure 5(b)). WWP2 expression was downregulated (*P* < 0.05, Figure 5(c)), and the Spearman's correlation of WWP2 expression was negatively correlated with IDD grade (*P* = −0.929, Figure 5(d)). Pearson's correlation between miR-328-5p and WWP2 gene expression was significantly negative (*R* = −0.92, *P* < 0.001, Figure 5(e)). Bax and Caspase3 expression was significantly increased in the degenerative nucleus pulposus tissues, while Bcl-2 was decreased (*P* < 0.05, Figure 5(f)).

### 3.6. MiR-328-5p Regulates WWP2 Expression and Promotes Apoptosis of NP Cells

**Figure 6:**
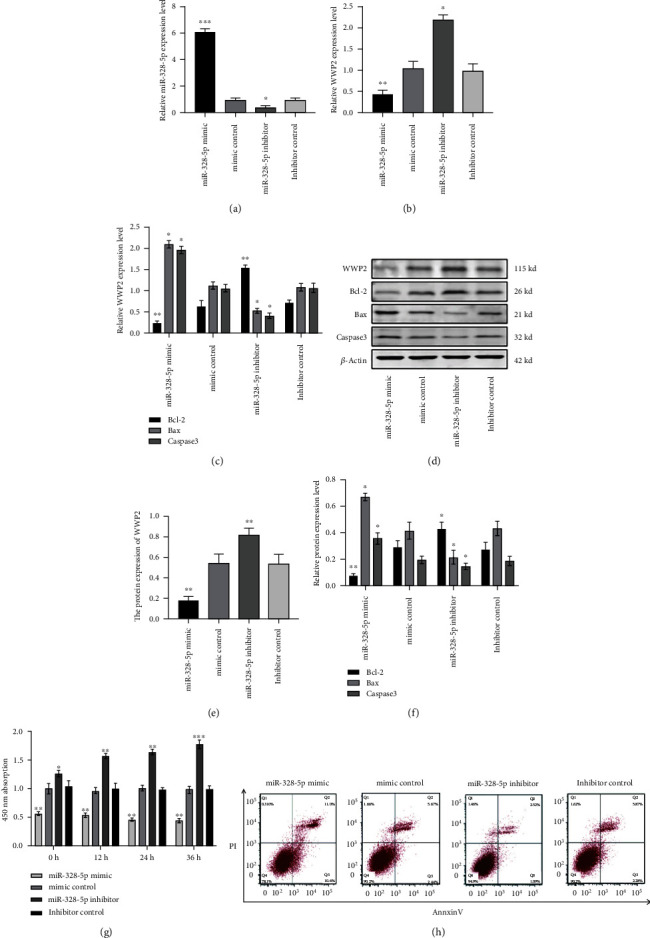
MiR-328-5p regulates WWP2 expression and promotes apoptosis of NP cells. (a) miR-328-5p gene expression. (b) WWP2 gene expression. (c) Bcl-2, Bax and Caspase3 gene expression. (d and e) WWP2 protein expression. (d and f) Bcl-2, Bax and Caspase3 protein expression. (g) NP cells proliferation. (h) NP cells apoptosis. ∗*P* < 0.05, ∗∗*P* < 0.01, ∗∗∗*P* < 0.001.

MiR-328-5p mimic induced miR-328-5p, Bax and Caspase3 gene expression obviously, while WWP2 and Bcl-2 was downregulated compared to the control group (Figures 6(a), 6(b), and 6(c)). miR-328-5p mimic significantly inhibited the protein expression of WWP2, Bcl-2 and promoted Bax and Caspase3 expression, while the miR-328-5p inhibitor obtained the opposite result (Figures 6(d), 6(e), and 6(f)). CCK8 assay confirmed that miR-328-5p mimic ignificantly inhibited the proliferation of NP cells (Figure 6(g)). Flow cytometry identified that the apoptosis of NP cells increased obviously after the addition of miR-328-5p mimic (Figure 6(h)). In conclusion, the above experimental results suggest that miR-328-5p can induce NP cells apoptosis.

### 3.7. MiR-328-5p Promotes the Apoptosis of NP Cells by Directly Targeting WWP2

**Figure 7:**
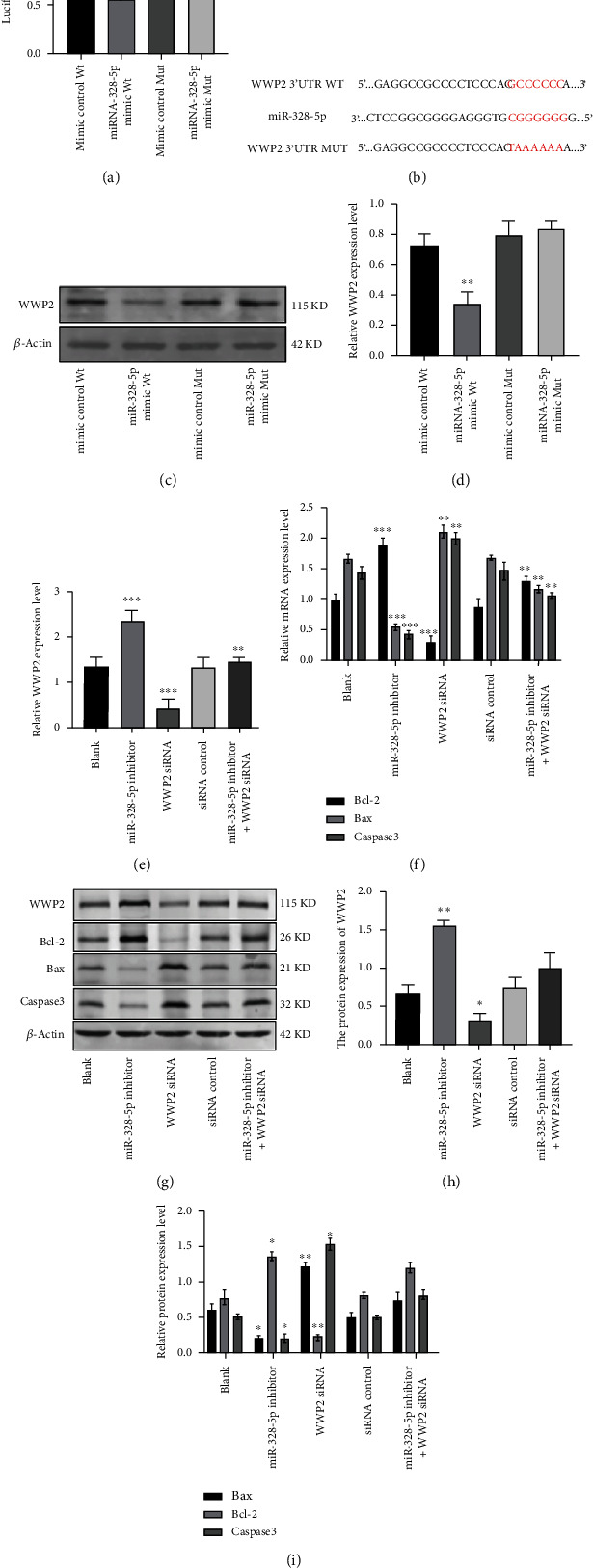
MiR-328-5p promotes the apoptosis of NP cells by directly targeting WWP2. (a) The cotransfection of WWP2 3′-UTR with miR-328-5p mimic. (b) Represent the corresponding sequence of Wt or Mut WWP2 3′-UTR plasmid and miR-328-5p. (c and d) WWP2 protein expression after the cotransfection. (e and f) WWP2, Bcl-2, Bax, and Caspase3 gene expression. (g, h, and i) WWP2, Bcl-2, Bax, and Caspase3 protein expression. ∗*P* < 0.05, ∗∗*P* < 0.01, ∗∗∗*P* < 0.001.

The luciferase activities were decreased after the cotransfection of wild type (Wt) WWP2 3′-UTR reporter plasmid and miR-328-5p mimic into NP cells (Figure 7(a)). The corresponding sequence of WWP2 3′-UTR plasmids Wt or Mut and miR-328-5p was enumerated (Figure 7(b)). WWP2 protein expression was downregulated after cotransfecting Wt and miR-328-5p mimic (Figures 7(c) and 7(d)). These results indicated that miR-328-5p directly regulates WWP2. And miR-328-5p inhibitor significantly upregulated WWP2 and Bcl-2 gene expression, while Bax and Caspase3 was downregulated compared to the control group (Figures 7(e) and 7(f)). And WWP2 and Bcl-2 protein expressions were upregulated, while Bax and Caspase3 expressions were downregulated posttransfection of miR-328-5p inhibitor (Figures 7(g)–7(i)). Transfection of the WWP2 siRNA into the miR-328-5p inhibitor group reversed these effects (Figures 7(g)–7(i)). In conclusion, these results confirm that miR-328-5p promotes the apoptosis of NP cells by directly targeting WWP2.

## 4. Discussion

Studies found that miRNA is mainly involved in IDD by regulating cell apoptosis and proliferation [41–44], inflammatory reaction [45–47], and extracellular matrix component degradation [48–51]. Some studies have found miR-328-5p regulated the proliferation and apoptosis of cancer cells [29–32]. Cao et al. found that silencing miR-328-5p significantly inhibited the proliferation of non-small cell lung cancer [29], and lncRNA RP5-916 L7.2 inhibited miR-328-5p expression and promoted the apoptosis of tongue squamous cell carcinoma cells [30]. Luo et al. speculated that miR-328-5p was a tumor suppressor, and they confirmed that miR-328-5p mimic decreased obviously the proliferation and cell cycle of breast cancer cells, and promoted apoptosis [31]. Overexpression of LINC00210 significantly decreased miR-328-5p expression and increased the proliferation and migration of non-small cell lung cancer cells [32]. As we know, WWP2 is essential for maintaining a stable cell cycle, though silencing of WWP2 reduces the rate of proliferation, and WWP2 regulates various cellular processes such as protein degradation, membrane protein endocytosis, apoptosis, and gene transcription. [52]. WWP2 accelerates the cell cycle and promotes tumor formation [34]. Downregulation of WWP2 decreased obviously lung adenocarcinoma proliferation [37]. Xu et al. found that WWP2 siRNA inhibited Bcl-2 expression by promoting Bax and Caspase7/8 to induce apoptosis of liver cancer cells [53]. Even more, overexpression of WWP2 could inhibit the apoptosis of human renal tubular epithelial cells by inducing Bcl-2 expressionand and inhibiting Bax expression [40]. However, the regulatory mechanism of miR-328-5p and its target gene WWP2 in IDD has not been reported.

In the study, through the analysis of miRNA and mRNA microarray database, we screened the target genes miR-328-5p and WWP2. The high expression of miR-328-5p, while low expression of WWP2 in a degenerative tissues by qRT-PCR. Surprisingly, the expression of miR-328-5p was positively correlated, while that of WWP2 negatively correlated with the degeneration grade of IDD. And we also identified that Bax and Caspase3 expression were upregulated, while Bcl-2 expression is downregulated. After miR-328-5p mimic transfected into nucleus pulposus cell, we found that the expressions of WWP2 and Bcl-2 were downregulated, while the expressions of Bax and Caspase3 were upregulated, and the same results were obtained by knocking down WWP2., and WWP2 siRNA could significantly reverse the effect of miR-328-5p inhibitor. MiR-328-5p mimic significantly inhibited the proliferation of nucleus pulposus cells compared with the control group by CCK8 assay. We also confirmed that miR-328-5p mimic increaed obviously the apoptosis of nucleus pulposus cells. WWP2 was identified as the direct target gene of miR-328-5p by bioinformatics. Compared with WWP2 Mut group, the luciferase activity of nucleus pulposus cells in WWP2 Wt group was significantly decreased, and WWP2 protein expression was also significantly downregulated.

In conclusion, these results strengthen our hypothesis that miR-328-5p regulated the prevalence and development of IDD by targeting WWP2. These results also indicate that miR-328-5p plays an essential role in regulating the proliferation and apoptosis of nucleus pulposus cells.

## 5. Conclusion

In conclusion, our results suggest that miR-328-5p is involved in the development of IDD by targeting WWP2 to induce the proliferation and apoptosis of nucleus pulposus cells. Furthermore, this study may provide a new reference for the diagnosis and treatment of IDD.

## Figures and Tables

**Table 1:** Clinical features of the study population.

Variable	Normal (*n* = 10)	IDD (*n* = 10)	*P* value
Age (years)	38.5 ± 3.5	41.6 ± 4.8	0.136^a^
BMI (kg/m^2^)	23.2 ± 1.0	23.5 ± 0.9	0.586^a^
Sex, *n* (%)			
(i) Female	4 (40)	3 (30)	0.639^b^
(ii) Male	6 (60)	7 (70)	

^a^Student's *t*-test. ^b^Two-sided *x*^2^-test. Data are presented as the mean ± SD or count (%).

## Data Availability

We confirm that this study data are available within the article or our supplementary materials. And the microarray data (such as Figure 1 and Figure 2) used in this study are available at the following link. The miRNA chip database is as follow:https://www.ncbi.nlm.nih.gov/gds/?term=GSE63492. The mRNA microarray database is as follows:https://www.ncbi.nlm.nih.gov/gds/?term=GSE34095.
